# Risk of Cardiovascular Events with Cyclin-Dependent Kinases 4 and 6 (CDK 4/6) Inhibitors among Patients with Advanced Breast Cancer: A Systematic Review and Network Meta-Analysis

**DOI:** 10.31083/j.rcm2411309

**Published:** 2023-11-09

**Authors:** Yi-Shao Liu, Kevin Dong, Chanhyun Park

**Affiliations:** ^1^College of Pharmacy, The University of Texas at Austin, Austin, TX 78712, USA

**Keywords:** CDK 4/6 inhibitors, endocrine therapy, advanced breast cancer, network meta-analysis, major adverse cardiovascular events (MACE), hypertension

## Abstract

**Background:**

Cyclin-dependent kinases 4 and 6 (CDK4/6) inhibitors have shown promising survival outcomes with additional treatments to the traditional endocrine therapy (ET) in patients with hormone receptor-positive (HR-positive) and human epidermal growth factor receptor type 2 negative (HER2–negative) advanced breast cancer (aBC). However, the head-to-head cardiovascular safety profile of these three agents (palbociclib, ribociclib, and abemaciclib) remains unclear. We summarized the incidence of major adverse cardiovascular events (MACE) and hypertension associated with the use of CDK4/6 inhibitor in randomized control trials (RCTs) and compared the risks of MACE and hypertension through network-meta analysis (NMA).

**Methods:**

A systematic search through PubMed and Cochrane Library was performed to identify phase III RCTs reporting cardiovascular safety data of CDK4/6 inhibitors in patients with aBC. We qualitatively synthesized the incidence of MACE and hypertension associated with CDK4/6 inhibitor use within on-treatment or placebo-controlled duration. A Bayesian NMA with random-effects models was performed, and pairwise comparisons between treatment options were presented by odds ratio (OR). The probability of each treatment arm’s relative ranking was reported using surface under the cumulative ranking curve (SUCRA) scores. A sensitivity analysis was conducted using the Mantel–Haenszel (MH) method.

**Results:**

Nine RCTs with four unique treatment arms and event(s) in at least one arm were included in the NMA. A total of 5218 patients were analyzed for MACE outcomes. The overall incidence of MACE in the CDK4/6 inhibitors+ET arm was 0.8%, while the endocrine therapy alone group was 0.4%. Abemaciclib+ET ranked the best in reducing the risk of MACE (SUCRA = 0.90) as compared to ET alone (SUCRA = 0.67, OR = 0.45, 95% credible interval (CI) = 0.07–2.82), palbociclib+ET (SUCRA = 0.25, OR = 0.09, 95% CI = 0.00–2.39) and ribociclib+ET (SUCRA = 0.17, OR = 0.08, 95% CI = 0.00–1.18). The findings were similar in the MH network. However, abemaciclib+ET (OR = 0.11; 95% CI = 0.02–0.81) had a significantly lower risk of MACE than ribociclib+ET in the MH network. No statistically significant differences in hypertension were shown among all comparisons.

**Conclusions:**

Abemaciclib+ET may have a lower risk of MACE for the treatment of aBC, while palbociclib+ET may reduce the risk of hypertension in this population. Our findings suggest a comparative cardiovascular safety trend among the three CDK4/6 inhibitors, but further research on direct comparisons is needed to guide treatment choice.

## 1. Introduction

Breast cancer is the most frequently diagnosed cancer in the United States (US) and worldwide with an increasing incidence rate of 0.5% each year [1, 2]. Hormone receptor-positive and human epidermal growth factor receptor type 2 negative (HR+/HER2–) is the most common subtype, with an approximate five-year survival rate of 90%. However, when diagnosed in late stages as advanced breast cancer (aBC), the five-year survival rate significantly decreases [3].

The cornerstone of treatment of aBC constitutes of endocrine therapy (ET): including aromatase inhibitors (such as letrozole, anastrozole, and exemestane), selective estrogen receptor (ER) modulators (such as tamoxifen), and ER down-regulators (such as fulvestrant). Unfortunately, the efficacy of these agents is limited by the presence of primary or acquired resistance [4, 5]. However, in recent years, it has been demonstrated that several solid tumors have direct modifications of genes that encode various proteins involved in cyclin-dependent kinases (CDK) and D-type cyclins activity and regulation [6]. As a result, small molecule inhibitors have been developed that target this mitogenic pathway, and three of them, known as cyclin-dependent kinases 4 and 6 (CDK4/6) inhibitors (palbociclib, ribociclib, and abemaciclib), are currently available for the treatment of aBC in combination with aromatase inhibitors or fulvestrant [6]. Three CDK4/6 inhibitors have been approved by the US Food and Drug Administration since 2015 as treatments in combination with ET for patients with HR+/HER– aBC, as informed by numerous randomized controlled trials (RCTs) [7, 8, 9, 10, 11, 12, 13]. In addition, several meta-analyses support this combined treatment which improves progression-free and overall survival of patients with aBC as compared to ET treatment alone [14, 15, 16, 17]. The American Society of Clinical Oncology guideline also recommends combined CDK4/6 and ET use [18, 19].

The use of CDK4/6 inhibitors, while providing clinical benefits, has raised concerns regarding cardiovascular events and cardiac toxicity [20, 21]. Significant QT prolongation was observed with ribociclib in the Mammary Oncology Assessment of LEE011’s (Ribociclib’s) Efficacy and Safety (MONALEESA) trials [9, 11, 13]. Furthermore, an updated review has reported that the use of palbociclib and ribociclib may be associated with an increased risk of thromboembolic events, based on data extracted from various clinical trials [22]. In addition, a prospective study has suggested that CDK4/6 inhibitors activate the inflammatory pathway, which may potentially lead to cardiovascular impairment [20].

Due to the heterogeneous safety profiles of different CDK4/6 inhibitors in relation to cardiovascular events, coupled with a lack of head-to-head trials comparing these drugs, we aimed to qualitatively synthesize the incidence of major adverse cardiovascular events (MACE) and hypertension associated with the use of CDK4/6 inhibitors reported in RCTs. Furthermore, this study conducted a network meta-analysis (NMA) to compare the MACE and hypertension risks associated with the use of CDK4/6 inhibitors.

## 2. Materials and Methods

This systematic review and network meta-analysis was reported in accordance with the Preferred Reporting Items for Systematic Reviews and Meta-Analyses (PRISMA) extension statement for systematic reviews, incorporating network meta-analyses for healthcare interventions.

### 2.1 Search Strategy 

A systematic search through PubMed and Cochrane Library was performed up until December 31, 2021 to identify phase III RCTs of CDK4/6 inhibitors with reported cardiac safety outcomes in aBC. In addition, each reference in the RCTs were manually reviewed to fully capture all the relevant studies that were not included in the above databases. The search strategy used key terms including breast cancer, cyclin-dependent kinase 4, CDK4, cyclin-dependent kinase 6, CDK6, ribociclib, palbociclib, and abemaciclib.

### 2.2 Study Selection and Evaluation 

The initial screening of title/abstracts was conducted independently by two reviewers (KD and YL) based on the inclusion and exclusion criteria. In the second screening, full-text articles were selected based on a full-text review. RCTs were eligible for inclusion based on the following criteria: (1) phase III RCTs of HR+/HER– aBC, (2) patients receiving either CDK4/6 inhibitors (palbociclib, ribociclib, and abemaciclib) plus ET or ET monotherapy, (3) with safety data of hypertension or MACE, which was defined as the composite endpoint of heart failure, myocardial infarction, cardiac arrest, cardiac failure, and stroke in our study [23], and (4) a restriction on English studies. Certain exclusion criteria were applied in our study. Firstly, studies that solely reported subgroup analyses derived from the main findings were excluded. Secondly, open-label studies with extended follow-up from the primary RCTs were also excluded. Additionally, studies that were not available in full-text format, including conference abstracts or reports, were not included in our analysis. Lastly, we excluded studies published prior to 2015 or those that contained investigation drug names.

### 2.3 Data Extraction and Quality Assessment 

Articles of interest were advanced to the data extraction stage after the two-stage screening process where two independent reviewers (KD and YL) identified the following key elements: author, year of publication, RCT name, journal of publication, countries, menopause status, the National Clinical Trial (NCT) number, line of therapy, drug treatment and comparator, age, number of patients (total and in each intervention), number of hypertension events, and number of major adverse cardiovascular events. Any discrepancies between the two reviewers were resolved by consensus and input from a third reviewer (CP). Moreover, the quality of the included studies was assessed by two reviewers (KD and YL) independently using the revised Cochrane risk of bias version 2 (RoB2) for RCTs [24].

### 2.4 Statistical Analysis 

A Bayesian network meta-analysis with random-effects models was performed using NetMetaXL (Canadian Agency for Drugs and Technologies in Health, Ottawa, Canada), which is a worksheet linked to WinBUGS 1.4.3 (Medical Research Council Biostatistics Unit, Cambridge, United Kingdom) [25]. In our network, we included RCTs that reported MACE or hypertension events in at least one treatment arm. Conversely, trials that reported zero events in all arms or did not report any MACE or hypertension events were excluded from the network analysis. Random-effects model was chosen due to the assumption of equal between-study heterogeneity variances across comparisons. Effect sizes were pooled using the Markov chain Monte Carlo approach with 10,000 iterations, and pairwise comparisons between treatment options were presented by odds ratio (OR) and 95% credible intervals (CIs). The intervention with the worst cardiovascular safety outcomes as determined by the NMA results was designated as the reference for the pairwise comparison. To compare the comparative safety of different treatment options regarding the risk of MACE or hypertension, we reported the probability of each treatment’s relative ranking using surface under the cumulative ranking curve (SUCRA) with a rankogram [26]. A SUCRA value approaching one indicates that a given treatment option has a higher likelihood of being ranked as the best. NetMetaXL also generated a plot that compares the posterior mean deviance of individual intervention pairs in the consistency and inconsistency models to detect loops in the treatment network where inconsistency may exist [25]. The Mantel–Haenszel (MH) approach was used in the R (Version 3.6.0, R Foundation for Statistical Computing, Vienna, Austria) software as a sensitivity analysis due to the sparse outcomes in our study [27].

## 3. Results

### 3.1 Characteristics of the Included Studies

**Fig. 1.**
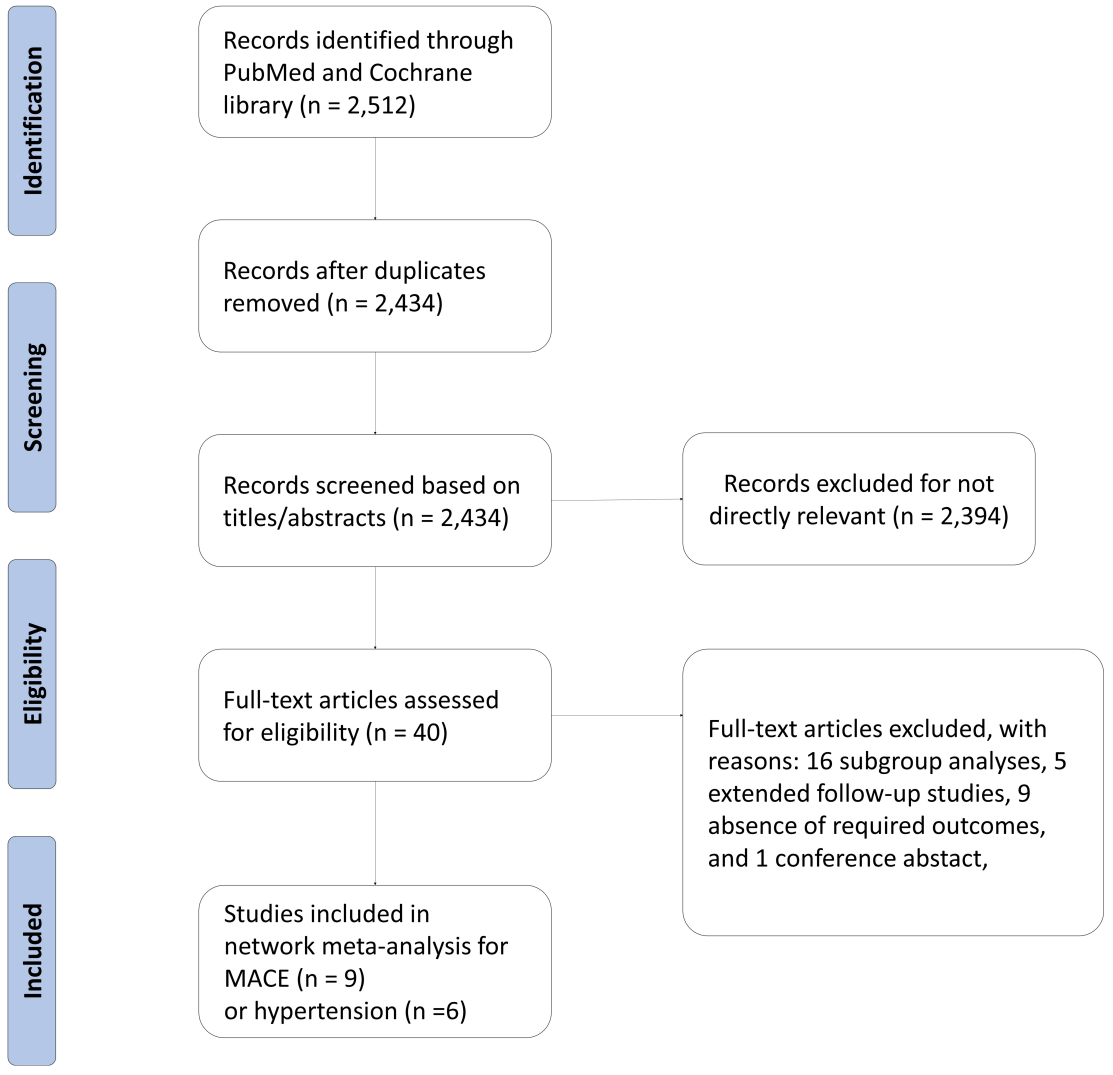
**Diagram flow of the study selection**. MACE, major adverse cardiovascular events.

**Fig. 2.**
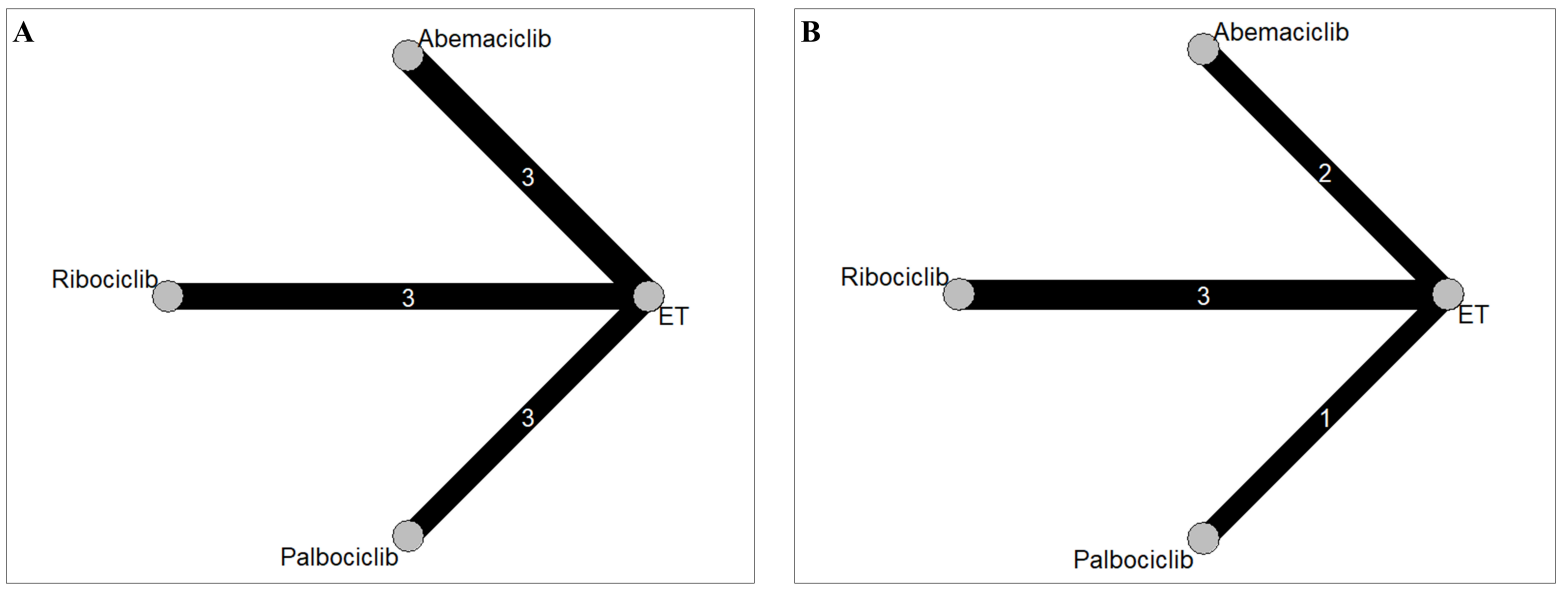
**Network geometry for (A) major adverse cardiovascular events and (B) hypertension**. The thickness of the lines corresponds to the number of trials comparing each treatment pair. ET, endocine therapy; Abemaciclib, Abemaciclib + ET; Ribociclib, Ribociclib + ET; Palbociclib, Palbociclib + ET.

Our network meta-analysis included a total of nine RCTs for MACE outcomes [7, 8, 9, 11, 12, 13, 28, 29, 30] and six RCTs for hypertension [7, 8, 9, 11, 13, 30], which involved 5218 and 3688 patients, respectively. The process of study selection was outlined in Fig. 1. Four eligible comparisons for the MACE and hypertension outcomes were presented in Fig. 2. Table 1 (Ref. [7, 8, 9, 11, 12, 13, 28, 29, 30]) provides a description of the baseline characteristics of the study population and the outcomes related to MACE and hypertension. A full list of excluded studies through the full-text review are provided in Appendix Table 2. Within the selected patients with HR+/HER– aBC, 3198 (61.0%) were administered CDK4/6 inhibitors in combination with ET, while 2020 (39.0%) received ET alone. Among those treated with CDK4/6 + ET, 960 (30.0%) were prescribed palbociclib, 1153 (36.1%) received ribociclib, and 1085 (33.9%) were given abemaciclib (Appendix Fig. 6).

**Table 1.** **Main characteristics of the studies included in the qualitative synthesis**.

Source [reference]	Journal	Country	Menopause Status	NCT Number	Line of Therapy	Drug treatment	${\mathrm{Age}}^{a}$	No. of ${\mathrm{patients}}^{a}$	No. of ${\mathrm{MACE}}^{a}$	No. of ${\mathrm{Hypertension}}^{a}$
PALOMA-2, [7]	*The New England Journal of Medicine*	17 countries (EU, NA, and Asia)	Post	NCT	1	Palbociclib + Letrozole	${\mathrm{62}}^{c}$	444	3	28
Finn *et al*., 2016				1740427		Letrozole	61	222	0	21
PALOMA-3, [28]	*The New England Journal of Medicine*	17 countries (EU, NA, and Asia)	Pre, Peri, and Post	NCT	2	Palbociclib + Fulvestrant	${\mathrm{57}}^{c}$	347	0	-
Turner *et al*., 2015				1942135		Fulvestrant	56	174	1	
PALOMA-4, [29]	*Annals of Oncology*	5 countries (Asia)	Post	NCT	1	Palbociclib + Letrozole	${\mathrm{53.8}}^{d}$	169	1	-
Xu *et al*., 2022				2297438		Letrozole	53.7	171	1	
MONALEESA-2, [9]	*The New England Journal of Medicine*	29 countries (EU, NA, SA, Asian, Australia, Africa)	Post	NCT	1	Ribociclib + Letrozole	${\mathrm{62}}^{c}$	334	1	48
Hortobagyi *et al*., 2016				1958021		Letrozole	63	334	0	49
MONALEESA-3, [11]	*The Journal of Clinical Oncology*	30 countries (EU, NA, SA, Asian, Australia)	Post	NCT	1	Ribociclib + Fulvestrant	${\mathrm{63}}^{c}$	484	9	48
Slamon *et al*., 2018				2422615		Fulvestrant	63	242	0	24
MONALEESA-7, [13]	*The Lancet Oncology*	30 countries (EU, NA, SA, Asian, Australia)	Pre and Peri	NCT	1	Ribociclib + Endocrine Treatment (AI/TAM)	${\mathrm{42.6}}^{d}$	335	3	29
Tripathy *et al*., 2018				2278120		Endocrine Treatment (AI/TAM)	43.7	337	0	23
MONARCH-2, [12]	*The Journal of Clinical Oncology*	20 countries (EU, NA, Asia, SA, Australia)	Post	NCT	1	Abemaciclib + Fulvestrant	${\mathrm{59}}^{c}$	446	3	-
Sledge *et al*., 2017				2107703		Fulvestrant	62	223	0	
MONARCH-3, [8]	*The Journal of Clinical Oncology*	23 countries (EU, NA, Asia, SA, Australia)	Post	NCT	1	Abemaciclib + NSAI (Anastrazole/Letrazole)	${\mathrm{63}}^{c}$	328	4	46
Goetz *et al*., 2017				2422615		NSAI (Anastrazole/Letrazole)	63	165	1	24
MONARCH Plus, [30]	*Therapeutic Advances in Medical Oncology*	4 countries (China, Brazil, India and South Africa)	Post	NCT	1	A: Abemaciclib + AI; AI	A: 56.4, ${\mathrm{55.5}}^{c}$	A: 207, 99	A: 2, 0	A: 14, 9
Zhang *et al*., 2020				2763566		B: Abemaciclib + Fulvestrant; Fulvestrant	B: 59.7, 58.2	B: 104, ${\mathrm{53}}^{b}$	B: 0, 0	B: 4, 0

^a^ Values are given for treatment group first and control group second. ^b^ There was more than 1 treatment group. The control group is second. ^c^ Median age. ^d^ Mean age. Abbreviations: AI, aromatase inhibitor; EU, Europe; MACE, major adverse cardiovascular events; NA, North America; NCT, National Clinical Trial; NSAI, nonsteroidal aromatase inhibitor; SA, South America; TAM, tamoxifen.

### 3.2 Risk of Bias in Included Studies

**Fig. 3.**
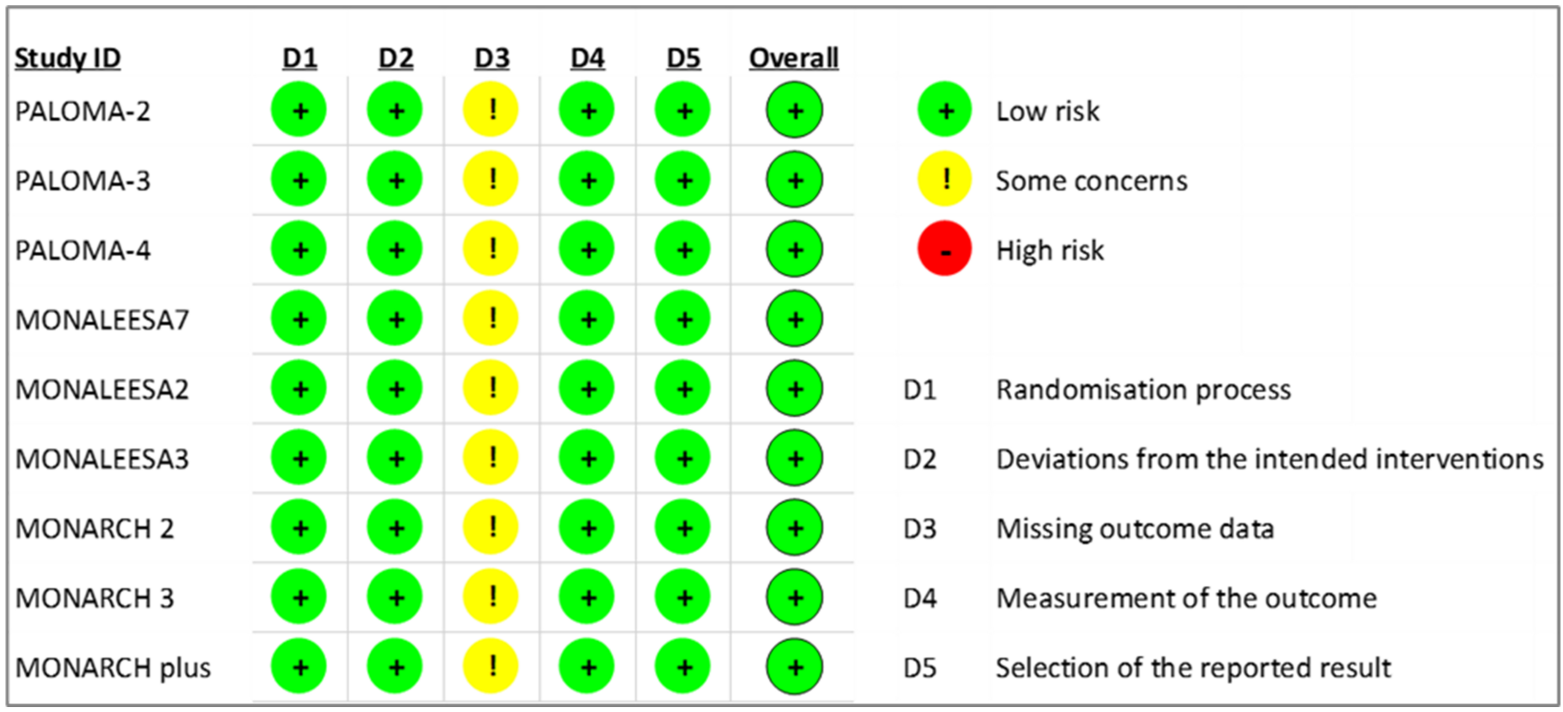
**Revised cochrane risk of bias for risk assessment on included randomized controlled trials**.

The RoB2 tool was used to assess the risk of bias in RCTs. While there were concerns about missing outcome data across all included studies in this systematic review [7, 8, 9, 11, 12, 13, 28, 29, 30], all RCTs were evaluated as having an overall low risk of bias based on the judgments of two assessors, as shown in Fig. 3.

### 3.3 Inconsistency Assessment

In the Appendix Fig. 7, most points were located in the upper left of the plots. Thus, the assessment of inconsistency plots between direct and indirect estimates for both outcomes suggests a low likelihood of inconsistencies impacting the results of the network meta-analysis.

### 3.4 Outcomes (MACE)

**Fig. 4.**
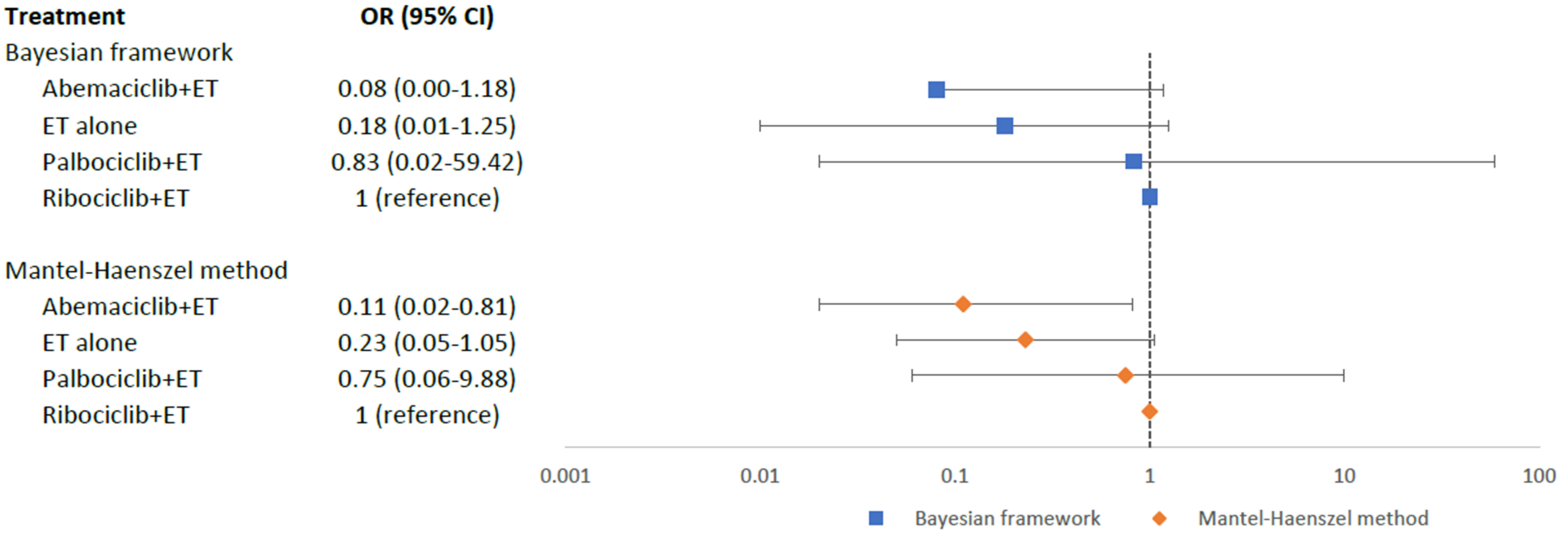
**Network meta-analysis plot for major adverse cardiovascular events of endocrine therapy and different CDK4/6 inhibitors**. Values are expressed as OR with 95% credible intervals. CI, credible intervals; ET, endocrine therapy; OR, odds ratio; CDK4/6, cyclin-dependent kinases 4 and 6.

The overall incidence of MACE in the CDK4/6 + ET group was 0.8%, whereas in the ET alone group, it was 0.4%. Based on our mixed comparisons among four intervention arms, no statistically significant differences were found in the occurrence of MACE among abemaciclib+ET (OR = 0.08; 95% CI = 0.00–1.18), ET alone (OR = 0.18; 95% CI = 0.01–1.25), and palbociclib+ET (OR = 0.83; 95% CI = 0.02–59.42), when compared to ribociclib+ET (Fig. 4). However, based on the relative ranking SUCRA, abemaciclib+ET (SUCRA = 0.90) was ranked as the best treatment with the least risk of MACE, followed by ET alone (SUCRA = 0.67), palbociclib+ET (SUCRA = 0.25); while ribociclib+ET (SUCRA = 0.17) was ranked the worst. Pairwise comparisons for MACE were displayed in a league table (Appendix Table 3).

In our sensitivity analysis using MH approach as shown in the forest plot (Fig. 4), abemaciclib+ET (OR = 0.11; 95% CI = 0.02–0.81) had a significantly lower risk of MACE as compared to ribociclib+ET. However, there were no statistically significant differences in the risk of MACE between the other two intervention arms as compared to ribociclib+ET: ET alone (OR = 0.23; 95% CI = 0.05–1.05) and palbociclib+ET (OR = 0.75, 95% CI = 0.06–9.88). Pairwise comparisons for MACE were displayed in a league table (Appendix Table 4), and the trend of the results was consistent to the Bayesian NMA.

### 3.5 Outcomes (Hypertension)

**Fig. 5.**
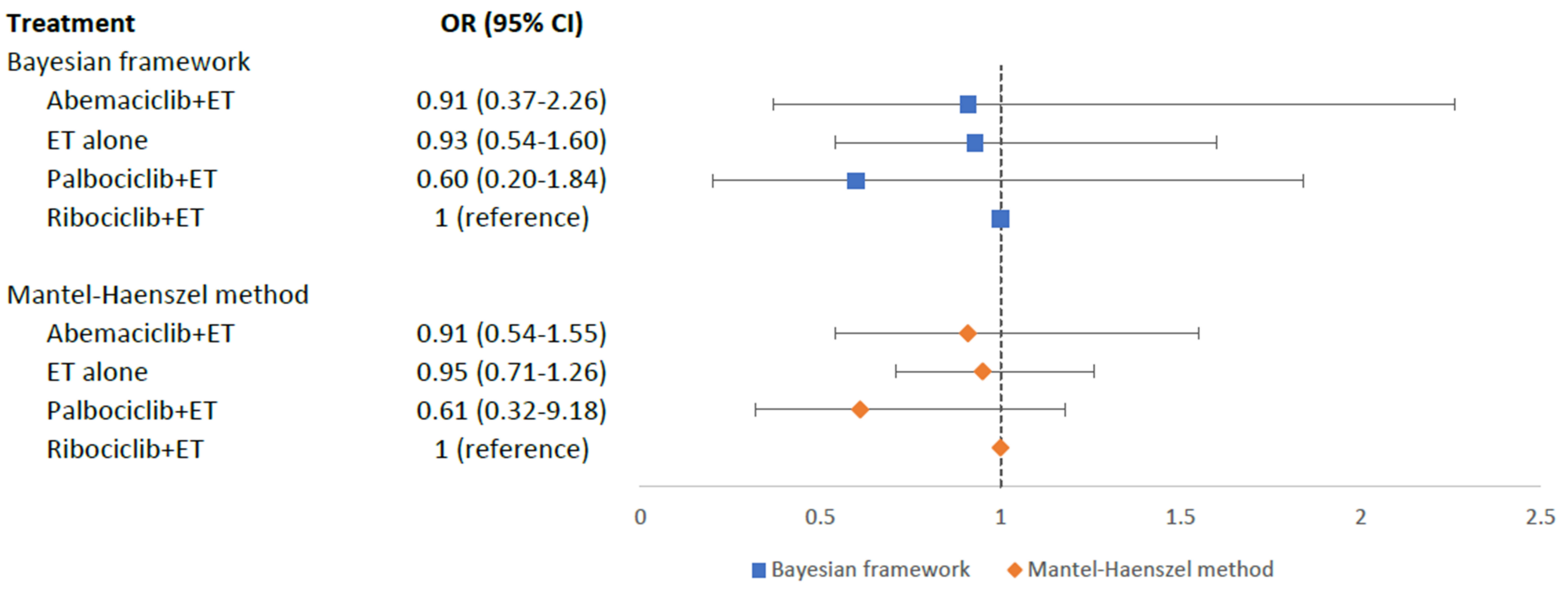
**Network meta-analysis plot for hypertension outcomes of endocrine therapy and different CDK4/6 inhibitors**. Values are expressed as OR with 95% credible intervals. CI, credible intervals; ET, endocrine therapy; OR, odds ratio; CDK4/6, cyclin-dependent kinases 4 and 6.

Hypertension risk was evaluated in six RCTs with 3688 aBC patients. As Fig. 5 showed, no statistically significant differences in the risk of hypertension were shown between different treatment arms and ribociclib+ET: palbociclib+ET (OR = 0.60, 95% CI = 0.20–1.84), abemaciclib+ET (OR = 0.91, 95% CI = 0.37–2.26), and ET alone (OR = 0.93, 95% CI = 0.54–1.60). According to SUCRA scores, palbociclib+ET (SUCRA = 0.83) was ranked the best with the least risk of hypertension, followed by abemaciclib+ET (SUCRA = 0.45), ET alone (SUCRA = 0.41), while ribociclib+ET (SUCRA = 0.30) was the worst treatment with the highest risk of developing hypertension. Pairwise comparisons for MACE in the Bayesian framework were displayed in the Appendix Table 5. The sensitivity analysis using MH method also confirmed these results (Fig. 5 and Appendix Table 6).

## 4. Discussion

The combination of CDK4/6 inhibitors and ET has been deemed an optimal treatment to prolong the survival outcomes of patients living with HR+/HER– aBC [15]. However, the negative impact of CDK4/6 inhibitors on the cardiovascular system has been investigated in some molecular studies [20, 31]. These studies have highlighted potential pathophysiological mechanisms such as increased vascular inflammation, left ventricular remodeling, and downregulation of the phosphatidylinositol 3‑kinase/protein kinase B (PI3/AKT) pathway [20, 31]. Despite the findings linking these effects to underlying cardiovascular disease development, a lack of sufficient evidence regarding the comparative cardiac safety of the three approved CDK4/6 inhibitors remains in the US. To address this gap, our systematic review and network meta-analysis confirms that the use of abemaciclib+ET could be associated with a lower risk of MACE, while ribociclib+ET had a less favorable safety profile regarding cardiovascular outcomes (including MACE and hypertension), based on mixed treatment comparisons. It is noteworthy that the sparse reporting of MACE events in the included phase III RCTs, may explain the lack of statistical significance in our study’s results. Nevertheless, both Bayesian and MH approaches indicated a similar trend of cardiac safety among the three CDK4/6 inhibitors in patients with HR+/HER– aBC.

Apart from ribociclib-induced QTc prolongation and non-cardiovascular related side effects, there is a lack of sufficient data available in other meta-analyses focusing on the incidence of cardiovascular events and outcomes associated with the use of these agents [21, 32, 33, 34]. The Palbociclib: Ongoing Trials in the Management of Breast Cancer (PALOMA) trials reported hematologic adverse events, primarily neutropenia, and leukopenia, as the most frequently observed adverse events with palbociclib [7, 34]. Conversely, neutropenia was the most commonly observed adverse event with ribociclib in the MONALEESA trials, where patients also showed QTc prolongation [9, 34]. Abemaciclib was associated with a higher incidence of gastrointestinal adverse events, which may be attributed to its distinct chemical structure and greater selectivity for CDK4 as compared to other CDK4/6 inhibitors [12, 35].

Our analysis showed that the combined use of abemaciclib plus ET was associated with a relatively lower risk of MACE. The included RCTs pertaining to abemaciclib also supported that the use of abemaciclib was not associated with a risk of MACE [8, 12, 30]. These findings could be explained by the unique structure of abemaciclib, which provides additional potency as a CDK9 inhibitor, compared to the other two agents that only exhibit reversible binding to CDK4/6 [33]. CDK9 is a kinase involved in cardiac hypertrophy [36], which suggests that abemaciclib possesses cardioprotective properties. The use of both CDK4/6 inhibitors and hormonal treatment could lead to endothelial injury and hypertension through vascular inflammation [20]. However, no statistically significant differences were detected in our analysis of hypertension incidence between the three agents. It is still unclear whether such inflammation is due to malignancy or drug-induced cardiotoxicity in cancer patients taking CDK4/6 inhibitors [20, 37]. Specific biomarkers associated with vascular inflammation in patients receiving CDK4/6 inhibitors have not been identified [38].

Our NMA indicated that ribociclib had a relatively higher risk of MACE compared to other agents. This may be attributed to its significantly higher likelihood of inducing QTc prolongation compared to the other two agents [9, 39]. Prolongation of the QTc interval is associated with sudden cardiac death and other heart-related adverse events [40]. Hence, the use of ribociclib in cancer patients with concurrent cardiovascular dysfunction should be carefully monitored and ribociclib should be avoided in patients with a QTc prolongation prior to initiating treatment.

In our study, the MH method was used as an alternative approach to testing the robustness of our findings, and the results were consistent with the trends of cardiac safety identified through the Bayesian NMA. However, the Bayesian approach exhibited wider CI than the MH method, although the point estimates were similar. Notably, a growing number of recent studies have discussed the use of Bayesian framework in the analysis of sparse outcomes, such as mortality and serious adverse events [41, 42]. The Bayesian model might be a concern as the prior distributions for the models’ parameters can strongly influence the results. In our study, a non-informative prior distribution was used in the random-effects Bayesian NMA, as there was no other external information established for our outcomes of interest. However, the impact of prior distributions on the posterior estimates of the model was believed to be more significant when dealing with sparse data [27]. Therefore, the utilization of both the Bayesian and MH methods for our network in this study adds further credibility to our findings.

Unfortunately, the RCTs analyzed in this network meta-analysis were underpowered to detect statistically significant differences in MACE and hypertension events among patients with aBC. This might result from a limited follow-up period and infrequent reporting of outcomes. For example, three RCTs were excluded due to a lack of reported hypertension events. More RCTs with a longer follow-up period and real-world studies involving a larger population are needed to validate the findings of our study. Furthermore, a future examination of patient subgroups with different comorbidities and age ranges would be valuable in helping clinicians determine which CKD4/6 inhibitors offer the most cardiovascular protection for patients with aBC.

### Study Limitations

The present network meta-analysis has some limitations to be considered. First, the analysis was limited by a relatively small number of studies and events of interest, which might lack the statistical power to detect differences in cardiovascular risks between intervention arms, thus potentially skewing the conclusions. Second, the lack of individual patient data represented another limitation. While the use of individual patient data in network meta-analysis would have led to more accurate estimates, such data were not accessible for the majority of the trials. Also, without the access to patient-level baseline characteristics, we were unable to further investigate the predisposing risk factors associated with MACE among patients with aBC included in the RCTs. Third, the heterogeneity of included RCTs was also a concern of the analysis. There were differences in patient population, lines of therapy, and age range across studies. For example, while PALOMA-2 enrolled postmenopausal patients with aBC [7], MONALEESA-7 included premenopausal women [43]. Furthermore, the baseline cardiovascular disease status varied in different RCTs, and this heterogeneity could have biased our study findings. Fourth, as mentioned earlier, some concerns were raised regarding the use of Bayesian models for sparse data. Thus, our study additionally enhanced the robustness of our findings by adopting a frequentist framework with the MH method as a sensitivity analysis. Finally, most of the RCTs were sponsored by pharmaceutical companies. This may have introduced risks of reporting bias and favorable findings for the funded agents.

## 5. Conclusions

Our study demonstrates that the combination of abemaciclib and ET led to a reduced risk of MACE in patients with HR+/HER2– aBC, while ribociclib plus ET was associated with a higher risk of cardiovascular events. Furthermore, the use of palbociclib+ET appeared to have a favorable safety profile with respect to hypertension. While most of the results in our analyses did not reach statistical significance, both Bayesian and MH network meta-analyses in our study supported the trend of comparative cardiovascular safety among the three CDK4/6 inhibitors. Given the growing importance of cardio-oncology, our findings could serve as a foundation for future research to further explore direct comparisons and aid in determining the optimal treatment choice.

## Figures and Tables

**Table 2.** **A full list of 31 excluded studies after the full-text review**.

Reasons for exclusion	Study
Subgroup analysis (N = 16)	1. Cristofanilli M, Turner NC, Bondarenko I, Ro J, Im SA, Masuda N,* et al*. Fulvestrant plus palbociclib versus fulvestrant plus placebo for treatment of hormone-receptor-positive, HER2-negative metastatic breast cancer that progressed on previous endocrine therapy (PALOMA-3): final analysis of the multicentre, double-blind, phase 3 randomised controlled trial. Lancet Oncol. 2016;17:425-39.
	2. Iwata H, Im SA, Masuda N, Im YH, Inoue K, Rai Y,* et al*. PALOMA-3: Phase III Trial of Fulvestrant With or Without Palbociclib in Premenopausal and Postmenopausal Women With Hormone Receptor-Positive, Human Epidermal Growth Factor Receptor 2-Negative Metastatic Breast Cancer That Progressed on Prior Endocrine Therapy-Safety and Efficacy in Asian Patients. J Glob Oncol. 2017;3:289-303
	3. Loibl S, Turner NC, Ro J, Cristofanilli M, Iwata H, Im SA,* et al*. Palbociclib Combined with Fulvestrant in Premenopausal Women with Advanced Breast Cancer and Prior Progression on Endocrine Therapy: PALOMA-3 Results. Oncologist. 2017;22:1028-38.
	4. O’Shaughnessy J, Petrakova K, Sonke GS, Conte P, Arteaga CL, Cameron DA,* et al*. Ribociclib plus letrozole versus letrozole alone in patients with de novo HR+, HER2- advanced breast cancer in the randomized MONALEESA-2 trial. Breast Cancer Res Treat. 2018;168:127-34.
	5. Verma S, Bartlett CH, Schnell P, DeMichele AM, Loi S, Ro J,* et al*. Palbociclib in Combination With Fulvestrant in Women With Hormone Receptor-Positive/HER2-Negative Advanced Metastatic Breast Cancer: Detailed Safety Analysis From a Multicenter, Randomized, Placebo-Controlled, Phase III Study (PALOMA-3). Oncologist. 2016;21:1165-75.
	6. Sonke GS, Hart LL, Campone M, Erdkamp F, Janni W, Verma S,* et al*. Ribociclib with letrozole vs letrozole alone in elderly patients with hormone receptor-positive, HER2-negative breast cancer in the randomized MONALEESA-2 trial. Breast Cancer Res Treat. 2018;167:659-69.
	7. Im SA, Mukai H, Park IH, Masuda N, Shimizu C, Kim SB,* et al*. Palbociclib Plus Letrozole as First-Line Therapy in Postmenopausal Asian Women With Metastatic Breast Cancer: Results From the Phase III, Randomized PALOMA-2 Study. J Glob Oncol. 2019;5:1-19.
	8. Masuda N, Inoue K, Nakamura R, Rai Y, Mukai H, Ohno S,* et al*. Palbociclib in combination with fulvestrant in patients with hormone receptor-positive, human epidermal growth factor receptor 2-negative advanced breast cancer: PALOMA-3 subgroup analysis of Japanese patients. Int J Clin Oncol. 2019;24:262-73.
	9. Mukai H, Shimizu C, Masuda N, Ohtani S, Ohno S, Takahashi M,* et al*. Palbociclib in combination with letrozole in patients with estrogen receptor-positive, human epidermal growth factor receptor 2-negative advanced breast cancer: PALOMA-2 subgroup analysis of Japanese patients. Int J Clin Oncol. 2019;24:274-87.
	10. Yardley DA, Hart L, Favret A, Blau S, Diab S, Richards D,* et al*. Efficacy and safety of ribociclib with letrozole in US patients enrolled in the MONALEESA-2 study. Clinical breast cancer. 2019;19:268-77. e1.
	11. Inoue K, Masuda N, Iwata H, Takahashi M, Ito Y, Miyoshi Y,* et al*. Japanese subpopulation analysis of MONARCH 2: phase 3 study of abemaciclib plus fulvestrant for treatment of hormone receptor-positive, human epidermal growth factor receptor 2-negative breast cancer that progressed on endocrine therapy. Breast Cancer. 2021;28:1038-50.
	12. Johnston S, O’Shaughnessy J, Martin M, Huober J, Toi M, Sohn J,* et al*. Abemaciclib as initial therapy for advanced breast cancer: MONARCH 3 updated results in prognostic subgroups. NPJ Breast Cancer. 2021;7:80.
	13. Kim JH, Im SA, Sim SH, Bananis E, Huang X, Kim HS,* et al*. Palbociclib Plus Fulvestrant in Korean Patients from PALOMA-3 With Hormone Receptor-Positive/Human Epidermal Growth Factor Receptor 2-Negative Advanced Breast Cancer. J Breast Cancer. 2021;24:97-105.
	14. Neven P, Johnston SRD, Toi M, Sohn J, Inoue K, Pivot X,* et al*. MONARCH 2: Subgroup Analysis of Patients Receiving Abemaciclib Plus Fulvestrant as First-Line and Second-Line Therapy for HR(+), HER2(-)-Advanced Breast Cancer. Clin Cancer Res. 2021;27:5801-9.
	15. Neven P, Rugo HS, Tolaney SM, Iwata H, Toi M, Goetz MP,* et al*. Abemaciclib plus fulvestrant in hormone receptor-positive, human epidermal growth factor receptor 2-negative advanced breast cancer in premenopausal women: subgroup analysis from the MONARCH 2 trial. Breast Cancer Res. 2021;23:87.
	16. Takahashi M, Tokunaga E, Mori J, Tanizawa Y, van der Walt JS, Kawaguchi T,* et al*. Japanese subgroup analysis of the phase 3 MONARCH 3 study of abemaciclib as initial therapy for patients with hormone receptor-positive, human epidermal growth factor receptor 2-negative advanced breast cancer. Breast Cancer. 2022;29:174-84.
Follow-up or Post Hoc Analysis (N = 5)	1. Hortobagyi GN, Stemmer SM, Burris HA, Yap YS, Sonke GS, Paluch-Shimon S,* et al*. Updated results from MONALEESA-2, a phase III trial of first-line ribociclib plus letrozole versus placebo plus letrozole in hormone receptor-positive, HER2-negative advanced breast cancer. Ann Oncol. 2018;29:1541-7.
	2. Johnston S, Martin M, Di Leo A, Im S-A, Awada A, Forrester T,* et al*. MONARCH 3 final PFS: a randomized study of abemaciclib as initial therapy for advanced breast cancer. npj Breast Cancer. 2019;5:5.
	3. Rugo HS, Finn RS, Diéras V, Ettl J, Lipatov O, Joy AA,* et al*. Palbociclib plus letrozole as first-line therapy in estrogen receptor-positive/human epidermal growth factor receptor 2-negative advanced breast cancer with extended follow-up. Breast Cancer Res Treat. 2019;174:719-29.
	4. Gelmon K, Walshe JM, Mahtani R, Joy AA, Karuturi M, Neven P,* et al*. Efficacy and safety of palbociclib in patients with estrogen receptor-positive/human epidermal growth factor receptor 2-negative advanced breast cancer with preexisting conditions: A post hoc analysis of PALOMA-2. Breast. 2021;59:321-6.
	5. Slamon DJ, Neven P, Chia S, Jerusalem G, De Laurentiis M, Im S,* et al*. Ribociclib plus fulvestrant for postmenopausal women with hormone receptor-positive, human epidermal growth factor receptor 2-negative advanced breast cancer in the phase III randomized MONALEESA-3 trial: updated overall survival. Ann Oncol. 2021;32:1015-24.
Absence of Required Outcomes (N = 9)	1. Durairaj C, Ruiz-Garcia A, Gauthier ER, Huang X, Lu DR, Hoffman JT,* et al*. Palbociclib has no clinically relevant effect on the QTc interval in patients with advanced breast cancer. Anticancer Drugs. 2018;29:271-80.
	2. Janni W, Alba E, Bachelot T, Diab S, Gil-Gil M, Beck TJ,* et al*. First-line ribociclib plus letrozole in postmenopausal women with HR+ , HER2- advanced breast cancer: Tumor response and pain reduction in the phase 3 MONALEESA-2 trial. Breast Cancer Res Treat. 2018;169:469-79.
	3. Diéras V, Harbeck N, Joy AA, Gelmon K, Ettl J, Verma S,* et al*. Palbociclib with Letrozole in Postmenopausal Women with ER+/HER2- Advanced Breast Cancer: Hematologic Safety Analysis of the Randomized PALOMA-2 Trial. Oncologist. 2019;24:1514-25.
	4. Fasching PA, Beck JT, Chan A, De Laurentiis M, Esteva FJ, Jerusalem G,* et al*. Ribociclib plus fulvestrant for advanced breast cancer: Health-related quality-of-life analyses from the MONALEESA-3 study. The Breast. 2020;54:148-54.
	5. Goetz MP, Martin M, Tokunaga E, Park IH, Huober J, Toi M,* et al*. Health-Related Quality of Life in MONARCH 3: Abemaciclib plus an Aromatase Inhibitor as Initial Therapy in HR+, HER2- Advanced Breast Cancer. Oncologist. 2020;25:e1346-e54.
	6. Harbeck N, Franke F, Villanueva-Vazquez R, Lu YS, Tripathy D, Chow L,* et al*. Health-related quality of life in premenopausal women with hormone-receptor-positive, HER2-negative advanced breast cancer treated with ribociclib plus endocrine therapy: results from a phase III randomized clinical trial (MONALEESA-7). Ther Adv Med Oncol. 2020;12:1758835920943065.
	7. Kaufman PA, Toi M, Neven P, Sohn J, Grischke EM, Andre V,* et al*. Health-Related Quality of Life in MONARCH 2: Abemaciclib plus Fulvestrant in Hormone Receptor-Positive, HER2-Negative Advanced Breast Cancer After Endocrine Therapy. Oncologist. 2020;25:e243-e51.
	8. Harbeck N, Iyer S, Turner N, Cristofanilli M, Ro J, André F,* et al*. Quality of life with palbociclib plus fulvestrant in previously treated hormone receptor-positive, HER2-negative metastatic breast cancer: patient-reported outcomes from the PALOMA-3 trial. Ann Oncol. 2016;27:1047-54.
	9. Sledge GW, Jr, Toi M, Neven P, Sohn J, Inoue K, Pivot X,* et al*. The Effect of Abemaciclib Plus Fulvestrant on Overall Survival in Hormone Receptor–Positive, ERBB2-Negative Breast Cancer That Progressed on Endocrine Therapy—MONARCH 2: A Randomized Clinical Trial. JAMA Oncology. 2020;6:116-24.
Unavailability of Full-text (N = 1)	1. Noguchi E, Yamanaka T, Yamamoto N, Chung C-F, Lu Y-S, Chang D-Y,* et al*. Palbociclib (P) plus tamoxifen (TAM) ± goserelin in women with hormone receptor-positive (HR+)/HER2-negative (HER2−) advanced breast cancer (ABC): Primary results of NCCH1607/PATHWAY, an Asian international double-blind randomized phase 3 trial. Journal of Clinical Oncology. 2023;41:LBA1068-LBA.

HER2–, human epidermal growth factor receptor type 2 negative; HR, homone receptor; PALOMA, Palbociclib: Ongoing Trials in the Management of Breast Cancer; MONALEESA, Mammary Oncology Assessment of LEE011’s (Ribociclib’s) Efficacy and Safety; TAM, tamoxifen; MONARCH, a series of clinical trials conducted to evaluate the efficacy and safety of the drug Abemaciclib in the treatment of various types of cancer.

**Table 3.** **League table of pair-wised comparisons among endocrine therapy and different CDK4/6 inhibitors in major adverse cardiovascular events using the Bayesian framework (with odds ratios and 95% credible intervals)**.

Abemaciclib+ET			
0.45 [0.07–2.82]	ET		
0.09 [0.00–2.39]	0.22 [0.00–2.97]	Palbociclib+ET	
0.08 [0.00–1.18]	0.18 [0.01–1.25]	0.83 [0.02–59.42]	Ribociclib+ET

Abbreviations: CDK4/6, cyclin-dependent kinases 4 and 6; ET, endocrine therapy.

**Table 4.** **League table of pair-wised comparisons among endocrine therapy and different CDK4/6 inhibitors in major adverse cardiovascular events using Mantel-Haenszel method (with odds ratios and 95% credible intervals)**.

Abemaciclib+ET			
0.50 [0.14–1.72]	ET		
0.15 [0.01–1.72]	0.31 [0.04–2.47]	Palbociclib+ET	
0.11 [0.02–0.81]	0.23 [0.05–1.05]	0.75 [0.06–9.88]	Ribociclib+ET

Abbreviations: CDK4/6, cyclin-dependent kinases 4 and 6; ET, endocrine therapy.

**Table 5.** **League table of pair-wised comparisons among endocrine therapy and different CDK4/6 inhibitors in hypertension outcomes using the Bayesian framework (with odds ratios and 95% credible intervals)**.

Abemaciclib+ET			
0.97 [0.62–1.54]	ET		
1.52 [0.71–3.22]	1.54 [0.84–2.78]	Palbociclib+ET	
0.92 [0.54–1.57]	0.95 [0.71–1.26]	0.61 [0.31–1.20]	Ribociclib+ET

Abbreviations: CDK4/6, cyclin-dependent kinases 4 and 6; ET, endocrine therapy.

**Table 6.** **League table of pair-wised comparisons among endocrine therapy and different CDK4/6 inhibitors in hypertension outcomes using Mantel-Haenszel method (with odds ratios and 95% credible intervals)**.

Abemaciclib+ET			
0.96 [0.62–1.51]	ET		
1.50 [0.71–3.14]	1.55 [0.86–2.80]	Palbociclib+ET	
0.91 [0.54–1.55]	0.95 [0.71–1.26]	0.61 [0.32–1.18]	Ribociclib+ET

Abbreviations: CDK4/6, cyclin-dependent kinases 4 and 6; ET, endocrine therapy.

**Fig. 6.**
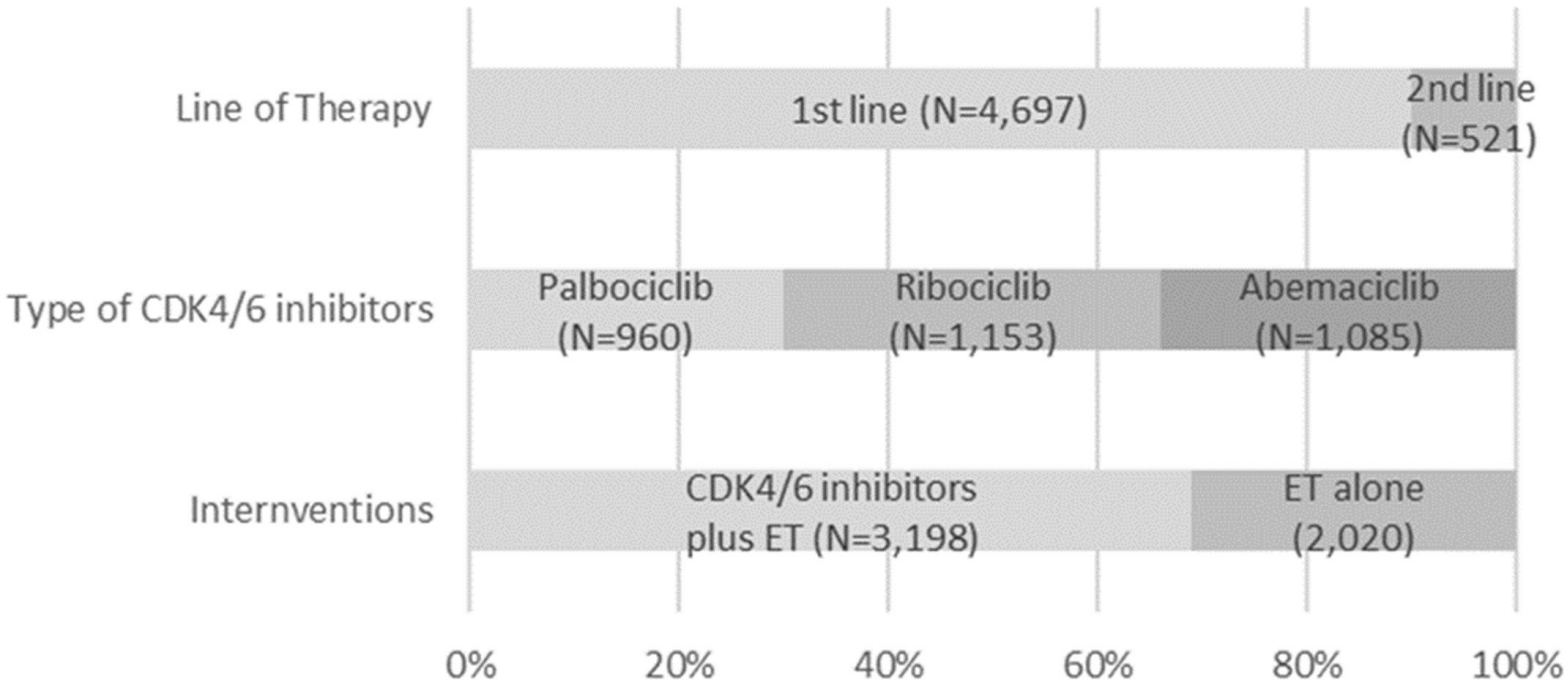
**A summary of key characteristics of 5208 patients involved in nine selected studies**. Abbreviations: CDK4/6, cyclin-dependent kinases 4 and 6; ET, endocrine therapy.

**Fig. 7.**
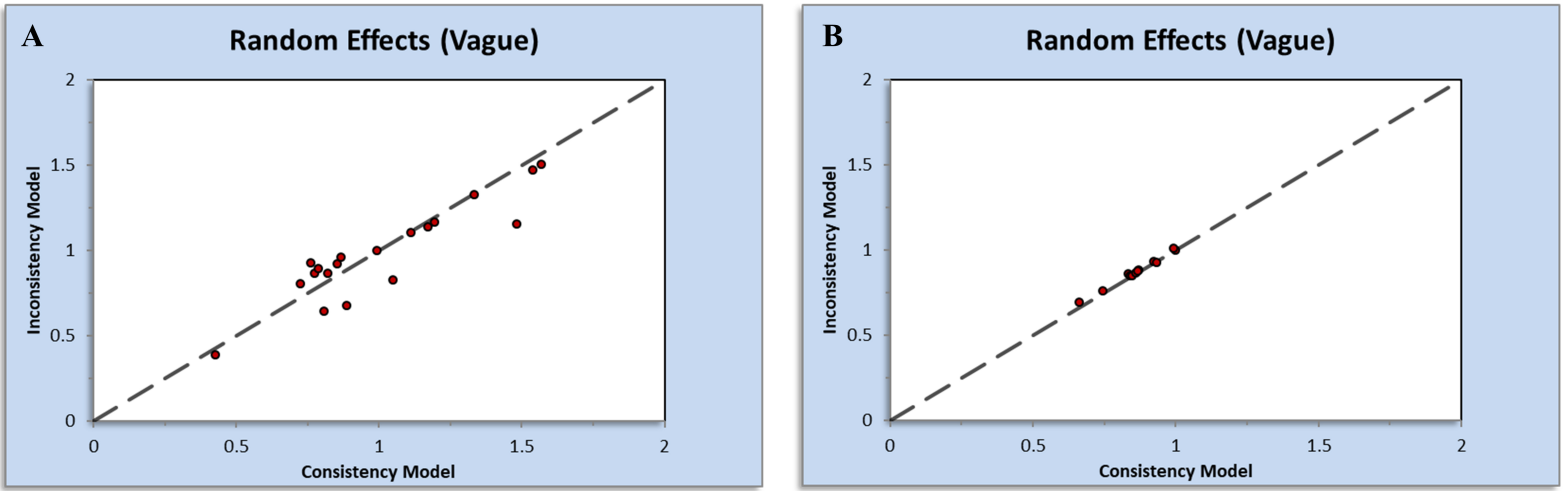
**Inconsistency plots generated by NetMetaXL for (A) major adverse cardiovascular events and (B) hypertension**.

## Abbreviations

aBC, advanced breast cancer; CDK4/6, cyclin-dependent kinases 4 and 6; CIs, credible intervals; ET, endocrine therapy; HR+/HER2–, hormone receptor-positive and human epidermal growth factor receptor type negative; MACE, major adverse cardiovascular events; MH, Mantel–Haenszel; NMA, network meta-analysis; OR, odds ratios; RCTs, randomized controlled trials; SUCRA, the surface under the cumulative ranking curve.

## Supplementary Material

Supplementary material associated with this article can be found, in the online version, at https://doi.org/10.31083/j.rcm2411309.

## Appendix

See Tables 2,3,4,5,6 and Figs. 6,7.
